# Does an activity based remuneration system attract young doctors to general practice?

**DOI:** 10.1186/1472-6963-12-68

**Published:** 2012-03-20

**Authors:** Birgit Abelsen, Jan Abel Olsen

**Affiliations:** 1 National Centre of Rural Medicine, University of Tromsø, Tromsø, Norway; 2 Department of Community Medicine, University of Tromsø, Tromsø, Norway

## Abstract

**Background:**

The use of increasingly complex payment schemes in primary care may represent a barrier to recruiting general practitioners (GP). The existing Norwegian remuneration system is fully activity based - 2/3 fee-for-service and 1/3 capitation. Given that the system has been designed and revised in close collaborations with the medical association, it is likely to correspond - at least to some degree - with the preferences of *current *GPs (men in majority). The objective of this paper was to study which preferences that young doctors (women in majority), who are the *potential entrants *to general practice have for activity based vs. salary based payment systems.

**Methods:**

In November-December 2010 all last year medical students and all interns in Norway (n = 1.562) were invited to participate in an online survey. The respondents were asked their opinion on systems of remuneration for GPs; inclination to work as a GP; risk attitude; income preferences; work pace tolerance. The data was analysed using one-way ANOVA and multinomial logistic regression analysis.

**Results:**

A total of 831 (53%) responded. Nearly half the sample (47%) did not consider the remuneration system to be important for their inclination to work as GP; 36% considered the current system to make general practice *more *attractive, while 17% considered it to make general practice *less *attractive. Those who are attracted by the existing system were men and those who think high income is important, while those who are deterred by the system are risk averse and less happy with a high work pace. On the question of preferred remuneration system, half the sample preferred a mix of salary and activity based remuneration (the median respondent would prefer a 50/50 mix). Only 20% preferred a fully activity based system like the existing one. A salary system was preferred by women, and those less concerned with high income, while a fully activity based system was preferred by men, and those happy with a high work pace.

**Conclusions:**

Given a concern about low recruitment to general practice in Norway, and the fact that an increasing share of medical students is women, we were interested in the extent to which the current Norwegian remuneration system correspond with the preferences of potential GPs. This study suggests that an existing remuneration mechanism has a selection effect on who would like to become a GP. Those most attracted are income motivated men. Those deterred are risk averse, and less happy with a high work pace. More research is needed on the extent to which experienced GPs differ along the questions we asked potential GPs, as well as studying the relative importance of other attributes than payment schemes.

## Background

GP remuneration systems are basically of three kinds: fee-for-service (FFS), capitation, and salary [1]. In practice, blended payment systems, including at least two of the three kinds, are widely used. FFS revenues may come from two different sources - a third party (public or private) insurer and patients' out of pocket payments. Additionally, a public purchaser may pay a lump sum to cover some of the fixed costs of the practice depending on national cost variations, as well as bonuses if the GP achieves some pre-specified targets [2].

Such complex payment mechanisms appear to be a common feature of many countries' primary health care systems [3]. In Norway, the vast majority of GPs work full time. Few, if any, would do part-time work in hospitals. Apart from a minority of the 7% who are salaried, Norwegian GPs have private practice and receive roughly 1/3 based on capitation paid by the municipalities and 2/3 FFS. These standard contracts do not include any performance elements. Capitation payment involves a flat rate per patient on the list. The FFS part is a mix of a fixed fee paid by patients, and variable fees paid by the federal government, depending on the duration of the consultation, on whether certain types of examinations and laboratory tests are initiated, and on whether or not the GP is a specialist in general medicine [4]. These intricate activity based payment contracts stand in sharp contrast to the salary based payments of doctors in hospitals and other parts of the health sector.

There are currently 5,000 GPs in Norway as compared to approximately 11,500 hospital doctors [5]. The average annual gross income of hospital doctors was NOK 770,000 (1€ = 7.75 NOK) in 2008 [6]. The comparable income of GPs is not easily accessible. However, information obtained from the Norwegian Medical Association refers to an average net profit (gross income) among self-employed GPs of NOK 1,030,000 in 2009. Even if this figure is not directly comparable due to their expenses for pension and sickness insurance, there is little doubt that GPs earn more than hospital doctors and that this is common knowledge among young entrants to the medical profession in Norway. There is no educational requirement beyond their internship to practice as a GP. However, 53.4% of GPs are specialists [5], which normally would take 6 years after internship to become.

Clearly, activity based systems may have behavioural effects on GPs' practice profiles: capitation rewards having many patients on the list, and FFS rewards consultations with many tests and prescriptions [7]. This is not surprising to economists, but still controversial among health care workers and politicians [8]. Theories of economic incentives are largely based on a hypothesis of self-interest, while theories on public service motivation and intra-occupational norms - including medical ethics - would predict more altruistic preferences among GPs.

Payment systems have selection effects: activity based systems attract more able workers and people who are relatively more income motivated and risk tolerant [9-11]. While an existing remuneration system might correspond with the preferences of current GPs who may have been attracted by it, or have adapted and learned to benefit from it, the system does not necessarily correspond with the preferences of the majority of young doctors who are the potential entrants to general practice. The aim of this paper is to explore i) the extent to which the current activity based remuneration system affect young Norwegian doctors' inclination to choose general practice as their career path, and ii) which remuneration system they would prefer if they were to work as GPs.

Fee-for-service can be traced back to a time when GPs were independent private practitioners, a role in which they may still perceive themselves despite the fact that the main bulk of their revenues now stem from the public purse. This paper is not concerned about the goodness of this system in terms of efficiency and equity considerations, but rather the extents to which it may affect recruitment of GPs.

The most recent health reform in Norway states a need for a large increase in the number of GPs [12]. In 2010 the share of women among GPs was 35.5%, while it was 61% among last year medical students [13]. The influx of women in the medical profession signals a pending gender shift in the future GP workforce. Given the fact that women are less attracted by performance related pay than are men [10], an activity based remuneration system might represent an impediment to GP recruitment. t is hypothesized that respondents' views on GP payment systems would reflect gender differences and underlying differences in personality traits in terms of risk attitude, income motivation, and preferred work pace. We would also assume that the sub-group who has experienced general practice as interns would differ from those who have no prior GP experience.

## Methods

A cross-sectional study was conducted in November - December 2010 among all last year medical students and all interns in Norway (n = 1,562). Medical training in universities in Norway is followed by 18 months' compulsory preliminary internship; 12 months in hospitals and 6 months in general practice. Contact information was provided by the four Norwegian medical faculties and the organisers of internship (local health authorities and county governor offices). The information letter included a web link to an online questionnaire. Two reminders were mailed. The survey was reported to the Privacy Ombudsman for Research in Norway in accordance with notification requirements.

The questionnaire design process was preceded by a qualitative study whereby five medical students and three interns had been interviewed about their preferences for various job characteristics. The interviews were recorded and transcribed, and the data analysis revealed different aspects and understandings concerning views on remuneration and income, providing us with some highly relevant background when formulating the survey questions.

The first question sought to reveal respondents' inclination to work as a GP: 'Which job would you wish to have in 10-15 years?' GP was listed among six alternatives to which multi-opted answering was possible.

After being informed (or reminded) about the activity based nature of the current Norwegian system, they were then asked: 'Do you consider this remuneration system to have any effect on your inclination to work as a GP?' Three alternative answers were listed: 1) it makes it more attractive; 2) less attractive, and 3) it is not important. Thereafter, they were asked 'If you were to work as a GP, which remuneration system would you prefer if you could choose?' The optional answers were: 1) salary; 2) activity based (the present system); 3) mix of salary and activity based (including an open space to fill in preferred salary percentage), and; 4) don't know. Answers to these two questions are sought explained by differences in respondent characteristics such as gender, personality traits regarding risk attitude, the importance of high income, preferred work pace, as well as their GP-experience and their inclination to work as a GP (see Table 1).

**Table 1:** Respondent characteristics, n = 831

Variable	Value	n	Percent
Sex	Male	340	41
	Female	490	59
Experience	Last year medical student	256	31
	Intern in hospital	284	35
	Intern in GP practice	291	34
Inclined to work as GP	Yes	437	53
	No	394	47
Risk attitude (index range: 6-32)	Risk averse (index < 12)	139	17
	Risk neutral (index [12-22])	540	65
	Risk seeking (index > 22)	144	18
"It is important for me to have a high income"	Do not agree (index 1-2)	67	8
	Some agreement (index 3-4)	421	51
	Strong agreement (index 5-6)	340	41
"I am happy with a high work pace"	Do not agree (index 1-2)	80	10
	Some agreement (index 3-4)	431	52
	Strong agreement (index 5-6)	317	38

The importance of a high income, and work pace tolerance, were measured using statements (see Table 1) with which respondents were asked to state their level of agreement on a six-point ordinal scale ranging from strong disagreement to strong agreement.

Risk attitude was measured by six items (see Table 2) from the Jackson Personality Inventory-Revised [14], adapted and validated by Pearson et al [15]. These items have been used in several studies of medical decision making [16-20]. The respondent scored all items on a Likert scale (1-6). These scores were added to an index ranging from 6 (very risk averse) to 36 (very risk seeking). Respondents who scored lower than 1 standard deviation (SD) below the mean were classified as risk averse, those who scored 1 SD above the mean were classified as risk seeking, while those who scored in the range mean ± 1 SD were classified as risk neutral.

**Table 2:** Distribution of responses to the risk attitude statements (Percent)

	Strongly disagree 1	2	3	4	5	Stongly agree 6	n
a. I enjoy taking risks	15.7	30.6	22.8	21.8	7.8	1.2	829
b. I try to avoid situations that have uncertain outcomes*	3.4	17.5	26.8	23.6	21.5	7.2	828
c. Taking risks does not bother me if gains involved are high	9.0	26.1	30.4	23.0	9.5	1.9	829
d. I consider security an important element in every aspect of my life*	0.8	5.2	12.0	26.3	36.1	19.4	825
e. People have told me that I seem to enjoy taking chances	26.6	31.0	21.7	12.5	6.7	1.5	822
f. I rarely, if ever, take risks when there is another alternative*	4.2	14.8	29.3	22.4	17.5	11.8	830

* In the construction of the risk attitude index, statement b, d and f were reversely recorded.

The data was initially analysed by frequency counts, means, medians and T-tests. One-way ANOVA was used to test for differences in respondent characteristics among the groups giving different answers to the two main questions analysed (see Tables 3 and 4). Multinomial logistic regression analysis [21,22] was then used to create profiles of respondents who considered the GP remuneration to be 1) positive and make general practice *more *attractive, or 2) negative and make general practice *less *attractive (see Table 5). The same type of analysis was used to create profiles of respondents who preferred 1) salary, or 2) activity based remuneration, if they were to work as GPs and was free to choose remuneration system. The independent variables were all included as dummies. SPSS version 17.0 was used to perform the statistical analyses.

**Table 3:** The existing remuneration system's influence on inclination to work as GP

	"Do you consider the GP remuneration system to have any effect on your inclination to work as a GP?"				
	**Not important**	**Positive effect**	**Negative effect**	**n**	

	47%	36%	17%	829	

*One-way ANOVA analyses:*		*Means*			*P - value*

Sex (1 = male)	0.30	0.58	0.34	827	< 0.001
Risk attitude index (6-32)	17.12	18.25	16.65	814	0.003
High income important (1-6)	3.85	4.71	4.08	826	< 0.001
Feel happy with a high work pace (1-6)	4.05	4.37	3.76	826	< 0.001
GP experience (1 = Yes)	0.27	0.36	0.49	828	< 0.001
Inclined to work as GP (1 = Yes)	0.53	0.58	0.41	829	0.007

**Table 4:** Preferred GP remuneration system

	"Which remuneration system would you prefer if you were to work as a GP and had a free choice?"					
	**Salary**	**Mix of salary and activity based**	**Activity based**	**I do not know**	**n**	

	**20%**	**48%**	**20%**	**12%**	**829**	

*One-way ANOVA analyses:*		*Means*				*p-value*

Sex (1 = male)	0.28	0.43	0.58		729	< 0.001
Risk attitude index (6-32)	16.15	17.71	18.43		713	< 0.001
High income important (1-6)	3.92	4.25	4.60		726	< 0.001
Feel happy with a high work pace (1-6)	3.80	4.13	4.52		727	< 0.001
GP experience (1 = Yes)	0.39	0.33	0.38		729	0.291
Inclined to work as GP (1 = yes)	0.46	0.53	0.58		729	0.084

Total response and response analyzed by single independent variables

**Table 5:** Multinomial logistic regression analysis of the existing GP remuneration system's influence on the respondents' inclination to work as GP

Independent variables	Positive effect Odds Ratio (95%CI) n = 293	Negative effect Odds Ratio (95%CI) n = 135
Sex		
0 = Female, 1 = Male	2.79 (1.97-3.94)***	1.11 (0.71-1.74)
Risk attitude		
Neutral	1	1
Risk averse	1.06 (0.65-1.74)	1.74 (1.05-2.89)*
Risk seeking	1.38 (0.88-2.16)	1.47 (0.82-2.66)
Important to have a high income		
Do not agree	1	1
Some agreement	4.27 (1.62-11.24)**	1.27 (0.63-2.54)
Strong agreement	13.33 (5.00-35.71)***	1.97 (0.95-4.10)
Feel happy with a high work pace		
Do not agree	1	1
Some agreement	1.29 (0.66-2.53)	0.50 (0.27-0.93)*
Strong agreement	1.97 (0.98-3.97)	0.43 (0.22-0.84)*
Experience		
0 = Student or hospital intern, 1 = Yes (GP intern)	1.76 (1.19-2.46)**	2.61 (1.72-3.97)***
Inclined to work as GP		
0 = No, 1 = Yes	1.56 (1.10-2.21)**	0.68 (0.45-1.04)
Cox & Snell R^2^	0.212	
Nagelkerke R^2 ^	0.244	

The reference category is: Remuneration system is not important (n = 385). *p < 0.05, **p < 0.01, *** *p *< 0.001

## Results

A total of 831 persons (53%) responded. The response rate differed across sub-groups: from 47% among hospital interns; 54% among last year medical students, and; 60% among GP interns. The gender balance among the respondents was identical to that in the sample invited to participate. The respondents differed in age between 23 and 53 years old. The mean and the median age was 28, and 83% were 30 years or younger. We do not know of any selection bias in terms of age. However, when we included age in the type of multinomial regression analyses reported in Tables 5 and 6, it had no significant effect. Country of graduation was asked for, but we do not know if there is any selection bias. However, as a group, respondents trained abroad did not answer significantly different from those trained in Norway. There were small differences in response rates among medical students between the four universities, in the range 48% and 57%. We tested for the possible existence of any clustering effect, or different norms, across the four sites, but we did not find any significant differences.

**Table 6:** Multinomial logistic regression analysis of preferred GP remuneration system

Independent variables	Salary Odds Ratio (95%CI) n = 163	Activity based Odds Ratio (95%CI) n = 160
Sex		
0 = Female, 1 = Male	0.59 (0.39-0.89)**	1.69 (1.15-2.49)**
Risk attitude		
Neutral	1	1
Risk averse	1.32 (0.82-2.12)	1.07 (0.60-1.89)
Risk seeking	0.94 (0.54-1.62)	1.00 (0.66-1.72)
Important to have a high income		
Do not agree	1	1
Some agreement	0.51 (0.27-0.95)*	0.94 (0.38-2.32)
Strong agreement	0.45 (0.23-0.85)*	1.65 (0.67-4.03)
Feel happy with a high work pace		
Do not agree	1	1
Some agreement	0.79 (0.44-1.43)	2.24 (0.89-5.65)
Strong agreement	0.57 (0.30-1.10)	3.94 (1.54-10.10)**
Experience		
0 = Student or hospital intern, 1 = Yes (GP intern)	1.29 (0.87-1.90)	1.31 (0.88-1.95)
Inclined to work as GP		
0 = No, 1 = Yes	0.72 (0.49-1.05)	1.50 (1.01-2.23)*
Cox & Snell R^2^	0.110	
Nagelkerke R^2^	0.128	

The reference category is: Mix of salary and activity based remuneration (n = 390).

Those who answered "I don't know" (n = 100) are excluded from the analysis.

*p < 0.05, **p < 0.01, *** *p *< 0.001

Table 1 shows respondents characteristics as well as their distributions of preferences concerning income, work pace and risk. Note the strong majority of women among young Norwegian physicians (59%). While 53% in the total sample were inclined to work as a GP, there was a significant difference in the sub-group of students (61%) vs. in the sub-group of interns with a GP-experience (44%), i.e. it seems that after experiencing what it is like to work as GP, a large proportion is deterred by this career path. However, the number 53% would grossly overestimate their inclination to end up as GPs. As multioptional answers were possible for the six listed alternatives, the average number of options ticked was 2. Only 13% had ticked GP career only.

Some 40% stated strong agreement that high income is important, the same as for being happy with a high work pace. The correlation between the two was statistically significant though quite weak (Spearman's r = 0.157, *p *< 0.01). The distribution of responses to the risk attitude statements is given in Table 2. The mean risk attitude index was 17 and the standard deviation 5. The mean risk attitude index differed significantly between men and women (19 vs. 16, *p *< 0.001)

### Does the current GP remuneration system affect recruitment?

Nearly half the sample did not think that the current system have any influence on their inclination to work as a GP. Among those who considered it to have an impact, twice as many were attracted than deterred (see Table 3). Not surprisingly, the characteristics of those who thought the current activity based system has a *positive *effect on their inclination to work as a GP, differed from those who thought the system has a *negative *effect. Table 5 proves this point. Those who think that the current remuneration system makes it *more *attractive to work as a GP are to greater extent men and those who think it is important to have a high income. Conversely, those who think that the current system makes general practice *less *attractive are risk averse, and those not happy with a high work pace. Interestingly, interns who have worked in general practice appear to have got a more conscious view on the effect of the remuneration system for their inclination to become a GP (see Table 3). They either like the current remuneration system or they dislike it. However, the degree of disapproval is much stronger than the degree of approval. As expected, the current remuneration system has a positive effect on those who had said they were inclined to work as a GP.

### Which remuneration system is preferred?

About half the sample preferred a mix of salary and activity based remuneration if they were free to choose (see Table 4). Those who opted for such a blended system were asked to state which percentage should be a fixed salary. The preferred percentage ranged between 25 and 80%, the mean was 57.6% and the median 50%. The relative support for salary only and for activity based only was the same, 20% for each. In other words, among potential entrants to general practice, only one in five prefer the current remuneration system. Table 6 shows that those who prefer this system are to greater extents men, and those who feel happy with a high work pace. Conversely, those who prefer a salary based system are to greater extents women, and those who do not think high income is important.

When comparing how much of the variations are explained in Table 5 vs. Table 6 the higher pseudo R^2 ^measures in Table 5 may reflect a more conscious response to the question on whether the existing remuneration system has a positive or negative effect on their inclination to work as a GP, while the subsequent question on which remuneration system they would prefer (Table 6) may be perceived as a more hypothetical choice. A related explanation can be found by noting that having experience as GP-intern came out strongly significant in Table 5 but not in Table 6.

## Discussion

It is widely believed that professionals hold high standards in how they should perform, and that financial incentives are important, but not sufficient to determine their behaviors [23]. It was therefore expected that young potential GPs are driven by a complex mix of financial and non-financial motives in their choice of career path. Nearly half the sample did *not *think that the existing activity based remuneration system had an effect on their inclination to work as a GP. However, given that this group had scored significantly lower on the statement 'High income is important to me', they may generally be less interested in how GPs happen to be paid.

The existing system *did *have a positive effect on those who think that high income is important and on men. Conversely, the system has a deterrent effect on risk averse people and those who are not happy with a high work pace. This finding that risk averse people are deterred by activity based remuneration is consistent with what theory would predict. The principal-agent theory focuses on both risks and rewards [24], and emphasizes that risk averse people dislike income risk associated with variable pay when output depends upon factors beyond their control [10,24].

Only one in five young doctors would prefer a fully activity based remuneration system. There are significantly more men in the group preferring an activity based system, while there are significantly more women preferring full salary. The gender difference may be linked to differences in values that lead women to select work settings that best facilitate a work-family balance [25]. Accordingly, young female doctors may perceive salary a better choice in a situation where they are more likely to work part time than men primarily in order to better balance work and family [26]. However, the desire for flexibility and a more manageable workload because of greater family responsibilities is preferred by young male doctors as well. The gender difference might also be driven in part by gender differences in risk attitude. The entrepreneurial position as self-employed which comes with the activity based remuneration system is known in general not to attract risk averse individuals [27,28]. As expected, a salary system was preferred by those who do *not *think that high income is important, while activity based remuneration was preferred by those who are happy with a high work pace. In theory the probability of preferring activity based remuneration is higher the more productive a worker is [10], and being happy with a high work pace might indicate a productive and more able worker.

The effect of having stated that general practice would be a preferred career path revealed some interesting indications: Given that one has at least some knowledge about the payment system involved in this career, one should not be surprised that approving this system makes one inclined to become a GP. On the other hand, while not significant, there is indication that the reverse is also true for one subgroup, suggesting that the existing system has a negative effect on some of those who were inclined to become GP.

These findings indicate that the choice of payment scheme has some *selection effects *on which personality traits are attracted and which are deterred. The current activity based scheme attracts people who think high income is important, which suggests that this group perceive general practice to be potentially more lucrative than other career paths. Income motivated GPs are likely to let their clinical practice be more influenced by own financial motives than are those who do *not *think high income is so important. Hence, a higher service provision under FFS may not only reflect an *incentive effect *but a *selection effect *as well, in that the system attracts a sub-group of doctors who are relatively more income focused and therefore more responsive to financial incentives (see also [10,11]). This could be seen as a problem reasoned in increased risk of physician-induced demand [7]. In a totally different perspective, more focus and promotion of the business elements of general practice, represented by features like the activity based remuneration system and self-employment, might be a way of attracting more men to medical education to dam the influx of women.

In the choice of payment schemes it was not explicitly stated which expected income levels, or income ranges, that would be associated with the payment schemes. However, we believe our respondents would have a fairly good idea of the income levels among GPs vs. salaried hospital physicians, and would implicitly acknowledge that the potential income range is higher under an activity based remuneration system. Clearly, preferences may change over time. Income may be less important when you are a student, but more important when you start to build a family. Follow-up surveys on these respondents are therefore of much interest. Certainly, there are important job attributes beyond payment schemes that determine the choice of becoming a GP. Their relative importance is a research topic in its own, and several studies are currently undertaken, e.g. in Denmark [29] and Australia [30].

We have suggested the existence of a possible selection effect. It might well be that an existing system corresponds with the preferences of experienced insiders, but 'The times they are a-changin' and women have different pay scheme preferences [10]. The current study provides new insights into the ways in which different personality traits among young doctors affect their preferences for GP payment systems. Certainly, further studies are required, particularly one that would compare the preferences of potential entrants to general practice with the preferences of older experienced GPs.

## Conclusions

Given a concern about low recruitment to general practice in Norway, and the fact that an increasing share of medical students is women, we were interested in the extent to which the current Norwegian remuneration system correspond with the preferences of potential GPs. Only 20% of young doctors would prefer the current remuneration system if they were to work as a GP. This study suggests that an existing remuneration mechanism has a selection effect on who would like to become a GP. Those most attracted are income motivated men. Those deterred are risk averse, and less happy with a high work pace. Concerns about low recruitment to GP practice call for further research on how these preferences translate into actual choices. Further research is also needed on the extent to which experienced GPs differ along the questions we asked potential GPs, as well as studies on the relative importance of other attributes than payment schemes.

## Competing interests

The authors declare that they have no competing interests.

## Authors' contributions

Both authors contributed to designing the survey, interpreting the results, drafting and revising the manuscript, read and approve the final manuscript. BA analysed the data.

## Pre-publication history

The pre-publication history for this paper can be accessed here:

http://www.biomedcentral.com/1472-6963/12/68/prepub
